# IL-17A Promotes the Migration and Invasiveness of Cervical Cancer Cells by Coordinately Activating MMPs Expression via the p38/NF-κB Signal Pathway

**DOI:** 10.1371/journal.pone.0108502

**Published:** 2014-09-24

**Authors:** Minjuan Feng, Yidong Wang, Kunlun Chen, Zhuoqiong Bian, Jinfang Wu, Qing Gao

**Affiliations:** 1 Department of Obstetrics and Gynaecology, Second Affiliated Hospital, School of Medicine, Xi’an Jiaotong University, Xi’an, P. R. China; 2 School of Medicine, Xi’an Jiaotong University, Xi’an, P. R. China; 3 Department 5 of Rheumatology, The Fifth Hospital of Xi’an City, Xi’an, P. R. China; National Cancer Center, Japan

## Abstract

**Objective:**

IL-17A plays an important role in many inflammatory diseases and cancers. We aimed to examine the effect of IL-17A on the invasion of cervical cancer cells and study its related mechanisms.

**Methods:**

Wound healing and matrigel transwell assays were used to examine the effect of IL-17A on cervical cancer cell migration and invasion by a panel of cervical cancer cell lines. The levels of matrix metalloproteinases (MMPs) and tissue inhibitor of metalloproteinases (TIMPs) were investigated using western blotting. The activity of p38 and nuclear factor-kappa B (NF-κB) signal pathway was detected too.

**Results:**

Here, we showed that IL-17A could promote the migration and invasion of cervical cancer cells. Further molecular analysis showed that IL-17A could up-regulate the expressions and activities of MMP2 and MMP9, and down-regulate the expressions of TIMP-1 and TIMP-2. Furthermore, IL-17A also activates p38 signal pathway and increased p50 and p65 nuclear expression. In addition, treatment of cervical cancer cells with the pharmacological p38/NF-κB signal pathway inhibitors, SB203580 and PDTC, potently restored the roles of invasion and upregulation of MMPs induced by IL-17A.

**Conclusion:**

IL-17A could promote the migration and invasion of cervical cancer cell via up-regulating MMP2 and MMP9 expression, and down-regulating TIMP-1 and TIMP-2 expression via p38/NF-κB signal pathway. IL-17A may be a potential target to improve the prognosis for patients with cervical cancer.

## Introduction

Cervical cancer is the second most common cause of cancer-related mortality in women worldwide [1] and is one of the only known cancers caused by a virus that can be sexually transmitted. Recent researches find that immune cells and their secreted cytokines can not only contribute to the elimination of cancer cells, but also provide a proper microenvironment for tumor development as well as promote tumor progression [2], during which the local tumor microenvironment and the function state of immune cells play important roles [3].

Interleukin 17A (IL-17A) is a pro-inflammatory cytokine, and has been found contributed to many chronic diseases. Recently, IL-17A has been also frequently found in many cancers such as ovarian cancer [4], breast cancer [5], gastric cancer [6], and hepatocellular carcinoma [3]. The role of IL-17A in the development and progression of these cancers remains controversial. Using animal model, some studies find that IL-17A inhibited tumor growth and metastasis through IFN-c producing NK and T cells [6], [7]. Other studies show that IL-17A promoted tumor growth and metastasis [8], [9]. The effect may be correlated with the induction of tumor promoting microenvironment at tumor site [10].

Tumor metastasis is the leading cause of mortality associated with cancer [11]. Cancer cells need to degrade the ECM and invade into the lymphatic and vascular systems for dissemination to distant sites [12]. In this process, proteases such as matrix metalloproteinase(MMPs), play important roles [12]. Production and activation of MMPs is dependent on various cytokines, including TNF-α and IL-1 secreted by tumor cells [13], [14], fibroblasts [15], [16] and macrophages [16]. Previous studies found that IL-17A could regulate MMPs, IL-1 and TNF in periodontitis [17], and found that IL-17 receptor deficiency results in impaired expression of IL-1 and MMP3/MMP9/MMP13 in rheumatoid arthritis [18], indicating that IL-17A also plays an important role in the regulation of MMPs. MAPK signal pathway and NF-κB play important roles in the regulation of the production and activity of MMPs [10], [19]. And many effects of IL-17A are correlated with MAPK signal pathway and NF-κB [10], [20], [21].

In our study, we found that IL-17A could increase cell motility and invasion by the up-regulation of MMP2 and MMP9 via activating p38-NF-κB signal pathway.

## Materials and Methods

### Ethics statement

This research was approved by the Ethics Committee of the Second Affiliated Hospital, School of Medicine, Xi’an Jiaotong University. All cervical cancer patient participants with tissue examination provided their written informed consent to participate in this study.

### Cervical cancer samples

Cervical cancer specimens for mRNA were obtained from 50 cervical cancer patients in the department of Obstetrics and Gynecology, Second Affiliated Hospital, School of Medicine, Xi’an Jiaotong University. All patients had consented to tissue collection at the time of surgery. And all of the specimens were diagnosed as the cervical squamous cancer by the department of pathology. Among them, 11 were from cervical biopsy and were not included in the statistical analysis of invasion depth and lymphatic metastasis. None of the patients had received chemotherapy, immunotherapy or radiotherapy prior to specimen collection. Clinical stage and histological classifications were based on the International Federation of Gynecology and Obstetrics (FIGO) classification system. Tissue samples were divided into two portions: one part was frozen in −80°C for RNA isolation and the left was used for pathological diagnosis.

### Cell lines and experimental reagents

The HeLa, C33A, and Caski cell lines were obtained from American Type Culture Collection (ATCC) and were maintained in Dulbecco’s modified Eagle’s medium (DMEM) supplemented with 10% fetal bovine serum at 37°C, 5% CO_2_. Anti-MMP2, MMP9, TIMP-1, TIMP-2, β-actin,p38 and p-p38 were obtained from Cell Signaling Technology (Beverly, MA). Anti-NF-κB-p50, p65/Rel A and Histone H1 antibodies were purchased from Santa Cruz Biotechnology (Santa Cruz, CA, USA). 3-(4, 5-Dimethylthiazol-2-yl)-2, 5-diphenyltetrazolium bromide (MTT) and all other chemicals were obtained from Sigma (St Louis, MO), unless otherwise indicated.

### Cell migration and invasion assay

Cell migration and invasion abilities were tested by wound healing and invasion assays. Cell migration was assessed by a wound healing assay. Cells were cultured in 6-well plate until confluent rate reach 70–80% and then treated with or without IL-17A (50 ng/mL). The cell layer was wounded using a sterile tip and the spread of wound closure was observed and photographed. Invasion assay was performed with 24-well BioCoat Matrigel Invasion Chambers (Becton Dicknson, Bedford, MA) according to the manufacturer’s instructions. After cultured in medium with or without IL-17A (50 ng/mL), cells were seeded onto inner well and number of cells that invaded through the Matrigel was counted.

### RNA isolation and real-time PCR analysis

Total RNA was extracted from cultured cells with TRIzol reagent (Invitrogen), and mRNA expression levels were measured by qRT-PCR using an iQ5 multicolor real-time PCR Detection System (Bio-Rad) with SYBR Premix EX. Reverse transcription was performed with the PrimeScript RT reagent Kit (Perfect Real Time; TaKaRa) according to the manufacturer’s instructions. For mRNA analysis, GAPDH mRNA levels were used as internal normalization control. Fold changes were calculated and normalized using the CT method. Primers used were as follows: GAPDH (GCACCGTCAAGGCTGAGAAC and TGGTGAAGACGCCAGTGGA); MMP1 (ACTCTGGAGTAATGTCACACCT and GTTGGTCCACCTTTCATCTTCA); MMP2 (CCGTCGCCCATCATCAAGTT and CTGTCTGGGGCAGTCCAAAG); MMP3 (AGTCTTCCAATCCTACTGTTGCT and TCCCCGTCACCTCCAATCC); MMP9 (GGGACGCAGACATCGTCATC and TCGTCATCGTCGAAATGGGC); MMP10 (CCCACTCTACAACTCATTCACAG and TCAGATCCCGAAGGAACAGAT); MMP13 (TGCCAGTGCCCTTAAATTCCA and CAACAGGGTCTCAAACCCCA); IL-17A (CAATCCCACGAAATCCAGGATG and GTGGAGATTCCAAGGTGAGG).

### Zymography

Cells were treated with IL-17A at 37°C for 24 h, and samples of conditioned media were collected. Appropriate volumes of the unboiled samples were separated by 0.1% gelatin-8% SDS-PAGE electrophoresis. After electrophoresis, the gels were washed twice in 2.5% Triton X-100 at room temperature for 30 min and then incubated in reaction buffer (10 mM CaCl2, 40 mM Tris-HCl and 0.01% NaN3, pH 8.0) at 37°C for 12 h. Coomassie brilliant blue R-250 gel stain was then used to stain the gel. The intensities of bands on the gels were calculated using an image analysis system (Bio-Rad Laboratories, Richmond, CA).

### Quantification of MMP-2 and MMP-9 proteins

Cells were seeded at a density of 1×10^5^ cells/ml into 6-well plates a day before the experiment. The cells were cultured in fresh DMEM medium supplemented with 1% FBS and parental cells were cultured in fresh DMEM medium supplemented with 1% FBS with or without IL-17A (50 ng/mL). After 48 h of incubation, cell supernatants were collected, and then MMP2 and MMP9 concentrations were quantified using the ELISA kits (Shanghai Westang Bio-Tech Co., Ltd., Shanghai, China).

### Western blotting analysis

1×10^6^ cervical cancer cells were suspended in 250 µl of lysis buffer (40mmol/l Tris-HCl, 1 mmol/l EDTA, 150 mmol/l KCl, 100 mmol/l NaVO3, 1% Triton X-100, 1 mmol/l PMSF, pH 7.5). The nuclear lysates were harvested with NucBuster Protein Extration Kit (Novagen, Germany) according to manufacturer’s instructions. The proteins (50 µg) were separated by 10% SDS-polyacrylamide gel electrophoresis and transferred onto PVDF membranes. The membranes were subsequently blocked in non-fat milk (5% in Tris-buffered saline with TWEEN-20, TBST) buffer at 37°C for 1 h to block non-specific binding and were then incubated overnight with antibodies against p38, p-p38, MMP2, MMP9, TIMP-1, TIMP-2, NF-κB-p50, p65/RelA, Histone H1 or β-actin in TBST containing 5% defatted milk at 4°C. Then second antibodies were incubated. The bands were detected with an enhanced chemiluminescence kit (Amersham, ECL Plus, Freiburg, Germany) and exposed by autoradiography. The densitometry analysis was performed using Image J software (GE Healthcare, Buckinghamshire, UK).

### Statistical analysis

All data are shown as the mean ± standard deviation and analyzed using SPSS 13.0 software (SPSS Inc., IL). Statistical significance was analyzed using the student’s t-test. *P*<0.05 was considered statistically significant.

## Results

### Expression of IL-17A is positively associated with metastasis of cervical cancer

IL-17A mRNA expression was measured in 50 cervical cancer tissues by real-time PCR. Association study was further applied to investigate the clinical significance of IL-17A expression in 50 cervical cancer specimens. The result showed that IL-17A expression did not correlate to patients’ age, FIGO stage, and tumor size, while IL-17A expression was significantly correlated to patients’ invasion depth and lymphatic metastasis status (*P*<0.01, students t-test, Table 1). These results indicated that IL-17A might play an important role in cervical cancer metastasis.

**Table 1:** Correlation of IL-17A expression with clinicopathological features in 50 cervical cancer patients.

Clinicopathological Features	Number	IL-17A (mean±SD)	*P* value
**Age**			>0.05
<45	22	4.52±1.52	
≥45	28	5.19±1.43	
**FIGO stage**			>0.05
I–IIa	40	4.77±1.39	
≥IIb	10	5.40±1.89	
**Tumor size(cm)**			>0.05
<2	29	4.60±1.33	
≥2	21	5.30±1.65	
**Differentiation stage**			<0.05
GI+GII	33	4.45±1.69	
GIII	17	5.76±1.82	
**Invasion depth** *			<0.01
<1/2	14	3.31±1.79	
≥1/2	25	8.54±1.98	
**Lymphatic metastasis** *			<0.01
No	30	2.24±1.1	
Yes	9	14.83±2.5	

*Partial data unavailable, statistics was done on the available data.

### IL-17A increased motility of cervical cancer cells

**Figure 1:**
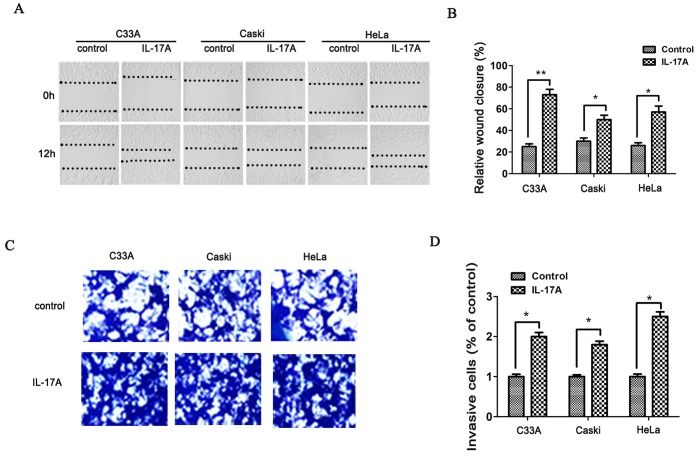
IL-17A promoted cervical cancer cells migration and invasion. (**A, B**) Compared with control group cells, IL-17A treated cervical cancer cells (HeLa, C33 A, and Caski) showed higher motility in a wound-healing assay. (**C**) By cell invasive assay, the effect of IL-17A on cell invasion was detected (magnification 100×). (**D**) Total invasive cell number in each chamber was summarized. Values are represented as means ± SD of three independent experiments performed in triplicate. * *p*<0.05 and ** *p*<0.01 compared with control group respectively.

Wound healing and matrigel invasion assays were conducted to further test the role of IL-17A on cervical cell motility. The results of wound healing show that migrations of HeLa, C33A and Caski cells were enhanced by IL-17A (Fig. 1A and B). Furthermore, transwell assay showed that treatment with IL-17A promotes cell invasion through the matrigel (Fig. 1C and D).

### IL-17A up-regulated MMP2 and MMP9 expression and down-regulated TIMP-1 and TIMP-2 expression in cervical cancer cells

**Figure 2:**
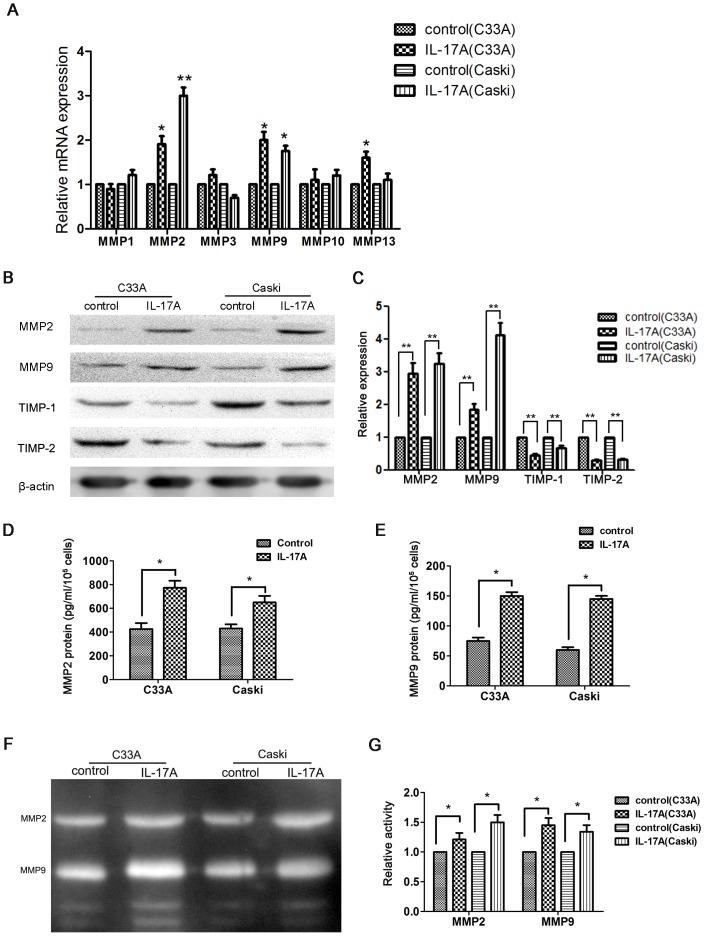
IL-17A upregulated MMPs expression and activity and downregulated TIMP expression. (**A**) MMPs expression was detected by real-time PCR analysis in the cervical cancer cells treated with and without IL-17A. (**B**) After treating with IL-17A for 24 hours, the expression of MMP2, MMP9, TIMP-1, and TIMP-2 were detected by western blot analysis in cervical cancer cells. (**C**) Quantification of the protein levels of MMP-2, MMP-9, TIMP-1, and TIMP-2. MMP2 (**D**) and MMP9 (**E**) concentrations in supernatants form cells treated with or without IL-17A were analyzed by ELISA. (**F**) The effects of IL-17A on the activities of MMP2 and MMP9 were analyzed by zymography assay. (**G**) Quantification of the activities of MMP-2 and MMP-9. Values are represented as means ± SD of three independent experiments performed in triplicate. * *p*<0.05 and ** *p*<0.01 compared with control group respectively.

As overexpression of MMPs play an important role in cancer metastasis [22], the role of IL-17A on MMPs expression in cervical cancer cell lines (C33A and Caski) was investigated. Expressions of MMP1, MMP2, MMP3, MMP9, MMP10, and MMP13 were detected by real-time PCR analysis between IL-17A treated and untreated cells. As shown in Fig. 2A, IL-17A increased the expression of both MMP2 and MMP9, indicating that the motility promoting role of IL-17A might be involved with extracellular matrix (ECM) remodeling.

MMP2 and MMP9 play important roles for cancer metastasis [12], and IL-17A can affect the expression of MMP2 and MMP9 [10]. TIMPs, the endogenous natural inhibitors of MMPs, can regulate the activity and expression of MMPs [23]. After treating with IL-17A, the expression of MMP2 and MMP9 proteins in cervical cancer cell lines (C33A and Caski) was increased, meanwhile the expression of TIMP-1 and TIMP-2 proteins was decreased(Fig. 2B and C).

### IL-17A increased the secretion and activity of MMP2 and MMP9

MMPs have the role of ECM degradation, and are strongly implicated in invasion and metastasis of malignant tumor cells [22]. In light of this, the expression of MMP2 and MMP9 in cell supernatants was analyzed by ELISA. As shown in Fig. 2D and E, the C33A cells secreted 425.55±49.35 pg/ml/10^5^ cells of MMP2 and 75.01±9.80 pg/ml/10^5^ cells of MMP9. IL-17A treatment significantly increased MMP2 levels to 773.12±60.12 pg/ml/10^5^ cells (*P*<0.05) and MMP9 levels to 148.89±11.18 pg/ml/10^5^ cells (*P*<0.05). Similar results were observed in Caski cells. Clearly, treatment with IL-17A remarkably promoted the expression of both MMP2 and MMP9.

Furthermore, the activity of MMP2 and MMP9 secreted by cervical cancer cells was examined by zymography assay. The results showed that IL-17A could significantly increase the degradation activity of MMP2 and MMP9 in C33A and Caski cell lines (Fig. 2F and G).

### IL-17A regulated MMPs expression and invasion of cervical cancer cells via activating p38/NF-κB signal pathway

**Figure 3:**
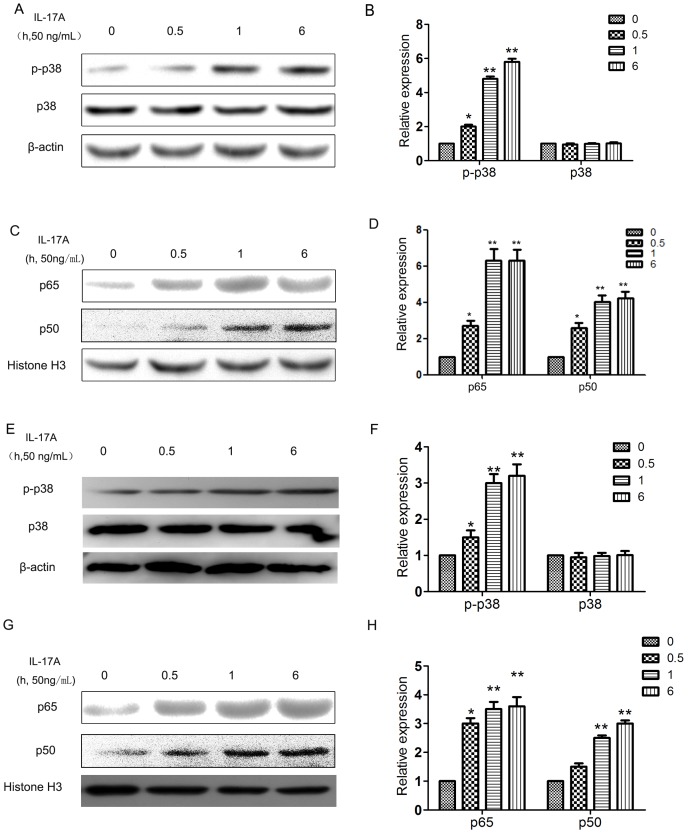
IL-17A can activate p38 and NF-κB signal pathways in cervical cancer cells. Expression of p38 and p-p38 were detected by western blot analysis in C33A(**A**) and Caski(**E**) cells treated with or without IL-17A for 24 hours. Quantification of the protein levels of p38 and p-p38 in C33A(**B**) and Caski(**F**). Values are represented as means ± SD of three independent experiments performed in triplicate. *p<0.05 and **p<0.01 compared with control group, respectively. Western blot analysis was used to detect nuclear p50 and p65 expression in C33A(**C**) and Caski(**G**) cells treated with IL-17A (50 ng/mL) at indicated time points. Quantification of the nuclear protein levels of p50 and p65 in C33A(**D**) and Caski(**H**) cells. Values are represented as means ± SD of three independent experiments performed in triplicate. * *p*<0.05 and ** *p*<0.01 compared with control group respectively.

**Figure 4:**
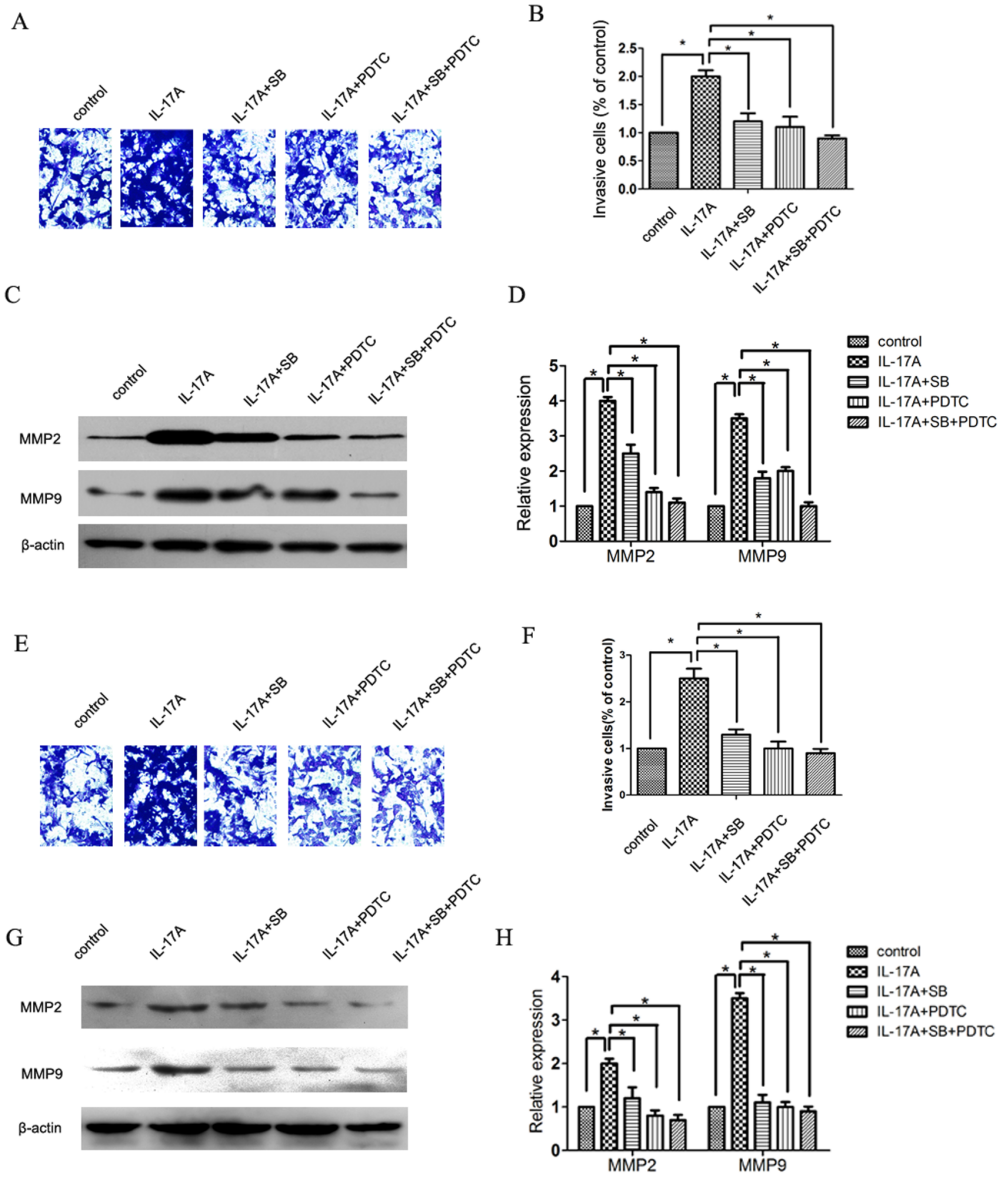
Effects of p38 inhibitor (SB203580, SB), NF-κB inhibitor (PDTC), and IL-17A on cell invasion and MMP2, MMP9 expression in cervical cancer cells. C33A(**A**) and Caski(**E**) cells were pretreated with SB (20 µM) and PDTC for 30 min, then incubated in the presence or absence of IL-17A (50 ng/mL) for 24 h. The cell invasive abilities were performed by Boyden chamber invasion assay. The percentage of invasive rate of C33A(**B**) and Caski(**F**) cells was expressed as a percentage of control. C33A(**C, D**) and Caski(**G, H**) cells were treated and then subjected to western blot to analyze the protein levels of MMP2, MMP 9. Values are represented as means ± SD of three independent experiments performed in triplicate. * *p*<0.05 and ** *p*<0.01 compared with control group respectively.

The p38 signal pathway plays important role in the invasion of cervical cancer cells. After treatment with IL-17A, the phosphorylation level of p38 was increased (Fig. 3A–B, E–F). However, the expression of total p38 was not affected. As NF-κB signaling pathway was reported to be a downstream target of p38 signaling, and was able to upregulate MMP2 andMMP9 expression, NF-κB/p50 and p65/RelA expression were also detected. We observed that treatment with IL-17A significantly increased the nuclear expression of both NF-κB/p50 and p65/RelA (Fig. 3 C–D, G–H). To further define the point in the p38/NF-κB signal pathway at which IL-17A regulates the invasion of cervical cancer cells, we treated cervical cancer cells with SB203580(a p38 inhibitor) and PDTC(a NF-κB inhibitor), and analyzed the invasive ability. Both SB203580 and PDTC can reverse the invasion increased by IL-17A (Fig. 4A–B, E–F). In addition, western blot analysis revealed that the pre-treatment of SB203580 and PDTC abrogated the upregulation of MMP2 and MMP9 induced by IL-17A (Fig. 4C–D, G–H), further demonstrating that IL-17A regulated MMPs expression and invasion of cervical cancer cells via activating p38/NF-κB signal pathway.

## Discussion

Substantial evidence indicates that certain cancer patients exhibit a generalized immunosuppressive status, but the inflammatory reaction at tumor site can foster tumor growth and progression [24], [25]. Persistent infection with human papillomavirus (HPV) is a necessary cause of cervical cancer [26]. HPV infections are common, and cervical cancer can be regarded as a rare complication of this common infection [27]. IL-17A is an important inflammatory cytokine in the development of many inflammatory diseases and it is also frequently detected in tumor microenvironment [8], [28], [29]. But up to now, little is known about the effect of IL-17A on cervical cancer progression. Souza and his co-workers have studied the correlation of the concentration of IL-17 on serum from patients and different grades of squamous intraepithelial lesions and invasive cervical carcinoma [30], not to mention the pro-metastatic and invasive effect of IL-17A on cervical cancer as well as its underling mechanism. Here, we revealed that IL-17A significantly promoted the invasive and metastatic ability of cervical cancer cells by regulating MMP/TIMP balance via activating the p38/NF-κB signal pathway.

In the present study, we found that IL-17A could enhance the migration and invasion abilities of cervical cancer cells. Previous research found that IL-17A could promote the migration and invasion abilities of human breast cancer and hepatocellular carcinoma cells [8], [10]. These results suggest that IL-17A is closely correlated with the invasion of cervical cancer cells. In order to clarify the related mechanisms, we investigated whether the promoting effect of IL-17A on cell invasion is through regulating expression of MMPs and TIMPs.

During the process of metastasis, cancer cells need to degrade the ECM and invade into blood or lymph vessels, and reach other tissues and organs, then generate new tumor. MMPs and TIMPs play important roles in degrading ECM [31]. MMP2 and MMP9 have been frequently detected to be over-expressed in solid tumors and associated with tumor invasion and metastasis [22], [32]. So we investigated the effect of IL-17A on the expression of MMP2 and MMP9. Results show that IL-17A can up-regulate the expression of MMP2 and MMP9. TIMPs act through the formation of a tight and noncovalent complex with their cognate enzymes and are able to affect the biological activities of MMPs [33], [34]. In present study, we found IL-17A could down-regualte the expression of TIMP-1 and TIMP-2. These results indicate that the promoting effect of IL-17A is correlated with the MMPs and their inhibitors.

The p38 signal pathway also plays important roles in the regulation of expression and activity of MMPs and TIMPs [35], [36]. Activity of p38 signal pathway can up-regulate the expression of MMP2 and MMP9 [10]. Previous study found that IL-17A can activate p38 signal pathway [37], [38]. To further clarify possible mechanism(s) of IL-17A in the promotion of cervical cancer cell invasion, we investigate the effect of IL-17A on the phosphorylation of p38. The results showed that IL-17A could up-regulate the phosphorylation level of p38. Results also showed that treatment of inhibitor of p38 reduced cell invasion significantly, accompanied by increased MMP2 and MMP9 protein expression, and decreased TIMP-1 and TIMP-2 protein expression, suggesting that the up-regulation role of IL-17A in MMP2 and MMP9 expression might be through the activation of p38 signal pathway. NF-κB has been found to be a key transcription factor in the regulation of MMP2 and MMP9 expression [10], [39] and IL-17A has been reported to be able to activate NF-κB signal pathway [40], [41], we next studied whether IL-17A could activate NF-κB signal pathway. The results showed that IL-17A could activate NF-κB, suggesting that the up-regulation role of IL-17A in MMP2 and MMP9 expression might be through the activation of NF-κB signal pathway.

In conclusion, the effect of IL-17A on cervical cancer cell invasion and metastasis may lead to the identification of new diagnostic markers and therapeutic targets.
